# Association of Neutrophil-to-Lymphocyte Ratio and Bloodstream Infections with Survival after Curative-Intent Treatment in Elderly Patients with Oral Cavity Squamous Cell Carcinoma

**DOI:** 10.3390/diagnostics13030493

**Published:** 2023-01-29

**Authors:** Chun-Hou Huang, Yu-Fu Chou, Tsung-Cheng Hsieh, Peir-Rong Chen

**Affiliations:** 1 Department of Nursing, Tzu Chi University, Hualien 97004, Taiwan; 2 Department of Otolaryngology, Hualien Tzu Chi Hospital, Buddhist Tzu Chi Medical Foundation, Hualien 970374, Taiwan; 3 School of Medicine, College of Medicine, Tzu Chi University, Hualien 970473, Taiwan; 4 Institute of Medical Sciences, Tzu Chi University, Hualien 97004, Taiwan

**Keywords:** oral cavity squamous cell carcinoma, elderly patients, neutrophil-to-lymphocyte ratio, bloodstream infection, survival

## Abstract

Patients with oral cavity squamous cell carcinoma (OSCC) undergoing curative-intent treatment may become immunocompromised. This study aimed to investigate the association of pretreatment sarcopenia, nutritional status, comorbidities, and blood-based inflammation prognostic biomarkers in bloodstream infection (BSI) with survival status in elderly patients with OSCC. Retrospective data were collected from 235 patients who were newly diagnosed with OSCC, were aged ≥ 65 years, had undergone curative-intent treatment, and were classified into either the BSI group or the no-BSI group within 6 months after surgery and/or adjuvant therapy initiation. Of the 235 elderly patients, 27 presented with BSI episodes. A preoperative high neutrophil-to-lymphocyte ratio (NLR) was a significant independent risk factor for BSI. BSI was not significantly associated with survival status. Ever betel nut chewing, hypoalbuminemia, and advanced tumor stage were associated with shorter overall survival. Moreover, a high NLR was an independent risk factor associated with disease-free survival. A high NLR was associated with BSI and resistance to curative-intent treatment. Pretreatment of NLR could act as an independent prognostic indicator and help inform treatment strategies for older patients with OSCC.

## 1. Introduction

According to the International Agency for Research on Cancer data, lip and oral cavity cancer was ranked sixteenth for incidence and mortality globally in 2020 [1]. Oral cavity squamous cell carcinoma (OSCC) accounts for > 90% of all oral cavity malignancies [2]. Regarding curative-intent treatment, based on the National Comprehensive Cancer Network (NCCN) guidelines, surgery is the main treatment for OSCC; moreover, it can be combined with radiotherapy (RT) and/or chemotherapy (Chemo) to treat patients with pathological adverse risk features [3]. Worldwide, there is a rapidly increasing number of elderly people diagnosed with cancer. However, older people are often restricted from clinical trials; accordingly, there is limited evidence regarding the efficacy of adopted treatment strategies [4].

Additionally, altered physiology, comorbidities, and functional and nutritional status impairment in elderly patients with OSCC increase the risk of treatment-related complications after aggressive curative therapy [5]. Accordingly, cancer treatment for older patients involves an increased risk of bacterial infectious complications. In the general population, surgical-site infections and subsequent complications after head and neck (HNC) surgery have been found to increase morbidity and the length of hospital stays [6]. However, there is limited evidence regarding post-treatment bloodstream infection (BSI) in the older population. As a result, improved knowledge of the predictive risk factors for BSI in older patients with OSCC is urgently needed.

Sarcopenia, nutritional status, and blood-based inflammatory biomarkers, including the lymphocyte-to-monocyte ratio (LMR), neutrophil-to-lymphocyte ratio (NLR), and platelet-to-lymphocyte ratio (PLR), could be clinical markers of systemic inflammation in patients with OSCC receiving anticancer treatment [7,8]. These clinical markers can be conveniently measured in routine clinical workups. However, the relationships between these significant clinical markers with BSI in older patients remain unclear. Therefore, there is a need to identify potential diagnostic and prognostic biomarkers in patients with BSI during and following treatment. This study aimed to examine the association of pretreatment risk factors and BSI with survival outcomes in elderly patients with OSCC after curative-intent treatment.

## 2. Materials and Methods

### 2.1. Study Design

This retrospective study was approved by the Research Ethics Committee of Hualien Tzu Chi General Hospital, Buddhist Tzu Chi Medical Foundation (IRB no: IRB109-292-B; 4 January 2021). This retrospective study included patients aged ≥ 65 years who were newly diagnosed with OSCC between January 2011 and January 2020 and treated at a single academic center through surgical resection with or without adjuvant therapy. The exclusion criteria were as follows: having metastasis and a history of malignancy, having an unresectable tumor, only receiving RT with or without Chemo, having a second primary tumor, and having incomplete follow-up information.

Patients with poor prognostic factors such as an advanced stage or pathologic features underwent postoperative adjuvant treatment and were managed in a multidisciplinary setting according to institutional protocols. The Chemo regimen was administered concurrently with RT as follows: (1) 20 mg/m^2^ cisplatin on days 1–5 at 3–4 week intervals with or without oral tegafur–uracil (each capsule contained 100 mg tegafur and 224 mg uracil), with 3–6 daily capsules in 2–3 divided doses; (2) 20 mg/m^2^ cisplatin with continuous infusion of 5-fluorouracil mg/m2 on days 1–5 every 3–4 weeks; and (3) oral tegafur-uracil, with 3–8 daily capsules in 2–4 divided doses. The only Chemo regimen involved oral tegafur–uracil, with 3–6 daily capsules in 2–3 divided doses for 12 months. The adjuvant regimen was chosen based on discussions between the physician and the patient, with appropriate adjustment of the dosage and duration.

All patients received cefmetazole as a prophylactic antibiotic 30 min before surgery and were managed for 7 postoperative days. Prophylactic antibiotics were prescribed at the discretion of the operating surgeon. During the adjuvant therapy period, patients with neutropenia did not receive antibacterial prophylaxis for preventing BSI.

### 2.2. Study Variables

Data regarding relevant demographic characteristics, the Charlson Comorbidity Index (CCI) [9], cancer sites, pathological stage, and treatment modalities were collected. Complete blood counts and albumin levels were measured using peripheral blood samples obtained within 1 perioperative week. The LMR, NLR, and PLR were calculated as the ratio of the lymphocyte-to-monocyte count, neutrophil-to-lymphocyte count, and platelet-to-lymphocyte count, respectively. The skeletal muscle area (SMA) was quantitatively analyzed at the C3 level of the preoperative computed tomography image. The C3 SMA was converted to estimate the SMA at the third lumbar vertebral (L3) level using a previously described equation [10,11]. Sarcopenia was indicated by an SMI < 43.2 cm^2^/m^2^, as previously described [12].

BSI occurrence from the date of operation with or without adjuvant therapy until 6 follow-up months was recorded. The date of BSI onset was defined as the date that positive culture findings were obtained. Post-treatment BSI was indicated by at least one positive blood culture for bacteria or fungi within 48 post-treatment hours. For coagulase-negative staphylococci and other common skin contaminants, BSI was indicated by at least two consecutive blood cultures positive for the same pathogen. For patients who had multiple blood cultures positive for the same organism, BSI events were considered independent if they occurred within intervals of ≥ 30 days. Regarding polymicrobial infections, each isolated causative organism was considered a separate BSI event.

The cutoff values for LMR, NLR, and PLR were determined through receiver operating characteristic curve (ROC) analysis [13] by calculating the area under the curve (AUC) for BSI. For overall survival (OS) and disease-free survival (DFS), the cutoff values for LMR, NLR, and PLR were determined using time-dependent ROC curves [14]. In all subsequent analyses, LMR, NLR, and PLR were stratified into high and low levels. OS was defined as the period from the date of diagnosis to the date of death, or censored at the date of the last follow-up for surviving patients. DFS was defined as the period from the date of operation to the date of disease progression or death.

### 2.3. Statistical Methods

Statistical analyses were performed using SPSS version 21.0 (IBM Corp, Armonk, NY, USA) and MedCalc Statistical Software version 19.4.1 (MedCalc Software, Ostend, Belgium). Descriptive statistics were provided for the BSI and no-BSI groups. Between-group comparisons of continuous variables were performed using independent t-tests or Mann–Whitney U tests. Between-group comparisons of categorical variables were performed using the Chi-square or Fisher’s exact test. Logistic regression models were used to evaluate the relationships between risk characteristics and BSI. ROC and time-dependent ROC curve analyses of blood-based inflammatory biomarkers were performed using EZR (Saitama Medical Center, Jichi Medical University, Saitama, Japan), which is a graphical user interface for R (The R Foundation for Statistical Computing, Vienna, Austria). Moreover, the cutoff value was determined using the Youden J index or as the value that maximized the sum of sensitivity and specificity. Univariate and multivariate Cox proportional hazards model analyses were performed to explore the association of the risk factors and BSI with survival outcomes; further, the results are presented as the hazard ratio (HR) with a 95% confidence interval (CI). Survival curves were obtained using the Kaplan–Meier method, and the log-rank test was used for comparison. Statistical significance was set at *p* < 0.05.

## 3. Results

### 3.1. Patient Characteristics

Initially, 268 patients aged more than 65 years, who were admitted for tumor resection with or without adjuvant therapy, were included in the present study. Patients with metastasis and a previous history of malignancy (*n* = 9), a second primary tumor (*n* = 6), and missing data (*n* = 8) and those who had only received RT with or without Chemo (*n* = 10) were excluded. Finally, 235 patients were identified to be eligible for inclusion in this study (men: 75.3%; median age: 71 years; interquartile range (IQR), 67.0–75.0 years). Supplementary Material Figure S1 illustrates the study flow chart. Table 1 summarizes the clinical characteristics of each group (BI group vs. no-BI group). Moreover, 109 (46%) and 126 (54%) patients received surgery alone and adjuvant treatment, respectively. Supplementary Material Table S1 presents details regarding the adjuvant Chemo regimen. Two patients could not complete the therapy due to treatment-related complications and toxicities.

There were no significant between-group differences in the proportion of patients aged ≥ 65 years, sex, alcohol assumption, smoking, betel nut chewing, CCI, nutritional status, tumor characteristics, treatment type, PLR, or LMR. The presence of BSI was associated with the prevalence of a higher NLR (median: 4.6; IQR: 2.0–5.8; *p* = 0.047). Table 2 summarizes the distribution of blood cultures. Overall, 38% (*n* = 19), 54% (*n* = 27), and 8% (*n* = 4) of the blood cultures grew Gram-positive bacilli, Gram-negative bacilli, and fungi, respectively. These pathogens were detected more within 1–3 months of BSI occurrence (80%, *n* = 40) than within 4–6 months of BSI occurrence (20%, *n* = 10). None of the patients died within 30 days of operation or adjuvant therapy.

### 3.2. Prognostic Utility of Inflammatory Biomarkers and Clinical Variables for BSI

**Figure 1:**
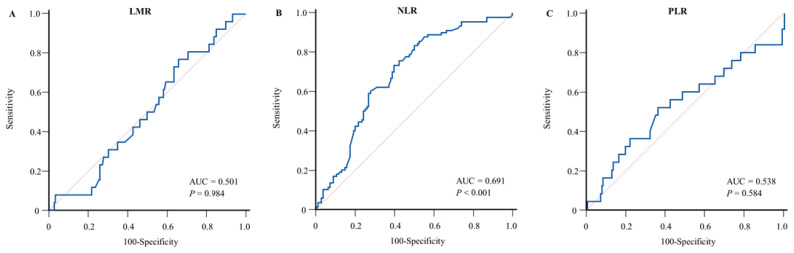
Receiver operating characteristic curves for determining the optimal cutoff value of preoperative systemic inflammatory markers for bloodstream infection. (**A**) Lymphocyte-to-monocyte ratio, (**B**) neutrophil-to-lymphocyte ratio, and (**C**) platelet-to-lymphocyte ratio.

ROC curves were calculated to select the optimal cutoff for LMR, NLR, and PLR with BSI as the primary endpoint. A high NLR was defined as having a value ≥ 5, with an AUC, sensitivity, and specificity of 0.69, 73.3%, and 60.4%, respectively. Figure 1 and Supplementary Material Table S2 present the cutoff values of LMR, NLR, and PLR as risk factors for BSI. Since there was no significant ROC curve for determining the optimal cutoff value of LMR and PLR, the cutoff value used median values of 4.0 and 135.6 for LMR and PLR, respectively. Logistic regression analysis revealed that only a high NLR was a significant risk factor for BSI (*p* < 0.001) (Table 3).

### 3.3. Survival Outcomes

**Figure 2:**
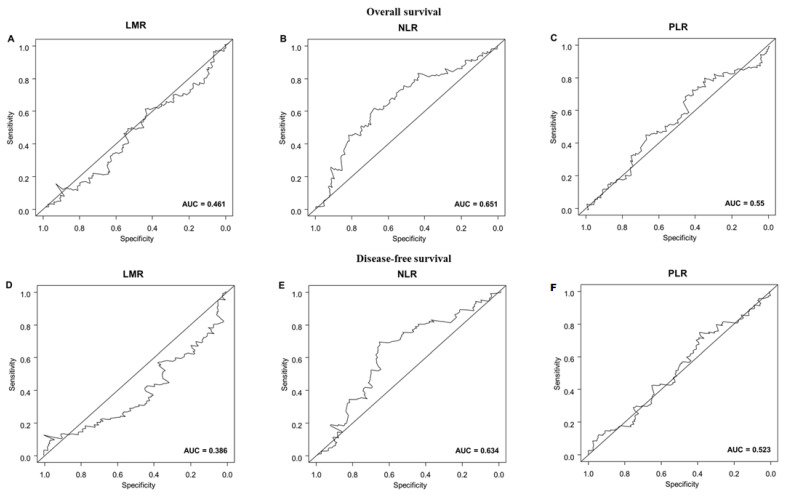
Time-dependent receiver operating characteristic curves for determining the optimal cut-off value of preoperative systemic inflammatory markers for overall survival ((**A**) Lymphocyte-to-monocyte ratio, (**B**) neutrophil-to-lymphocyte ratio, and (**C**) platelet-to-lymphocyte ratio) and disease-free survival ((**D**) Lymphocyte-to-monocyte ratio, (**E**) neutrophil-to-lymphocyte ratio, and (**F**) platelet-to-lymphocyte ratio).

**Figure 3:**
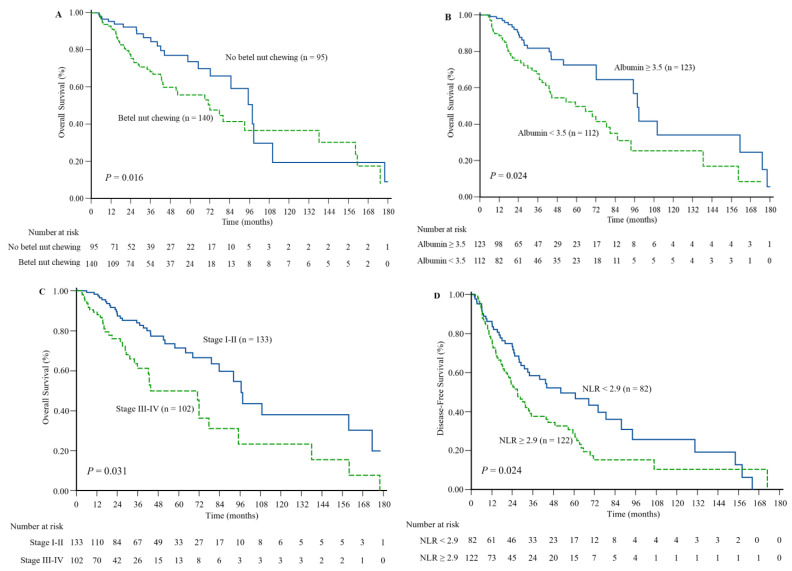
Kaplan–Meier estimates of survival outcomes. Prediction of overall survival based on betel nut chewing (**A**), albumin status (**B**), and tumor stage (**C**). Prediction of disease-free survival based on neutrophil-to-lymphocyte ratio (NLR) (**D**).

The median follow-up time after diagnosis was 42 months (range: 6–186 months). The OS and DFS were 31.4% and 54%, respectively. Figure 2 and Supplementary Material Table S3 present the cutoff values of LMR, NLR, and PLR as factors for OS and DFS. Table 4 and Table 5 present the results of univariate and multivariate Cox regression analyses of the clinical risk factors for OS and DFS, respectively. Significant variables and inflammatory biomarkers in the univariate analysis were fitted into the multivariate Cox regression model. Multivariate analysis revealed that ever betel nut chewing (*p* = 0.016), hypoalbuminemia (*p* = 0.024), and advanced tumor stage (*p* = 0.031) were significantly associated with OS. Moreover, a high NLR showed an independent significant association with shorter DFS (*p* = 0.024). The other variables were not significantly associated with DFS. Figure 3 presents the results of the Kaplan–Meier curve analysis and log-rank testing for patient ever betel nut chewing (no vs. yes, *p* = 0.016), albumin level (<3.5 vs. ≥3.5, *p* = 0.024), and tumor stage (I-II vs. III-IV, *p* = 0.031), with respect to OS. A higher NLR (<2.9 vs. ≥2.9, *p* = 0.024) was associated with a shorter DFS.

## 4. Discussion

In the present study, among 235 patients with OSCC aged ≥ 65 years, 27 (11.5%) developed BSI while receiving curative-intent treatment. BSI occurrence was associated with a higher NLR. Additionally, advanced tumor stage, ever betel nut chewing, and hypoalbuminemia were independent risk factors for shorter survival. Further, a higher NLR showed an independent significant association with a shorter DFS. The current study findings demonstrated that a high pretreatment NLR is a risk factor for BSI and recurrence events.

The incidence of post-treatment infection in patients with OSCC is approximately 30% [15,16]. Most studies have focused on surgical site infections in the general population, with only a few considering BSIs in elderly patients with OSCC. In the present study, the most common Gram-negative bacilli associated with BSIs were Acinetobacter baumannii and Pseudomonas aeruginosa, while the most common Gram-positive coccus was Staphylococcus aureus, which is consistent with previous findings by Tjoa et al. [6] and Jensen et al. [15]. Moreover, almost four out of five BSI episodes occurred within the first 3 months. Gram-negative bacilli often comprise the oral flora of patients and are involved in the etiology of postoperative pneumonia in HNC patients [17,18]. Staphylococcus aureus is the most common causative pathogen during RT [15]. Therefore, the present study findings confirm Balagopal et al. [19]’s reports that antibiotics against common pathogens should be included in the empiric antibiotic regimen, including cefoperazone/sulbactam for suspected systemic infections in older patients with OSCC undergoing curative-intent treatment.

In the general population, NLR is a strong negative, independent prognostic factor in patients with OSCC [20,21]. During the last decade, there has been increasing attention on the relationship between a high NLR and BSI in non-cancer patients [22,23]. The current study found that a high NLR was associated with the risk of BSI in older patients with OSCC after curative-intent therapy. Neutrophils have a very complex functional phenotype and release pro-inflammatory cytokines, including interleukin-1β, interleukin-6, and tumor necrosis factor-α, which are associated with negative outcomes [22,23,24]. During sepsis, neutrophils release neutrophil extracellular traps to bind pathogens. However, excessive neutrophil levels with concurrent sparsity of regulatory lymphocytes, including B-cells and T-cells, result in lymphocyte depletion, which leads to an unbalanced inflammatory response [25,26]. In addition, for older patients, there might be more significant impairment of adaptive immunity.

A high NLR is indicative of increased systemic inflammation; accordingly, the present study findings suggest that the inflammatory response may contribute to BSIs during curative-intent treatment in older patients with OSCC. Therefore, a high NLR due to inflammation could be associated with a poor prognosis in the present study. The NLR has shown diagnostic utility for BSIs, with moderate sensitivity and specificity. NLR can be obtained only via routine work-up and can decrease the additional cost. This indicates that compared with other biomarkers, the NLR can better reflect underlying immune function in peripheral blood, which is in agreement with the reports of previous studies [22,27]. A recent study by Jensen et al. [15] on BSIs in HNC patients who underwent curative-intent CRT reported that advanced age, tumor stage, and performance status were significantly correlated with an increased risk of BSI. However, this previous study did not include blood-based inflammatory biomarkers; moreover, the primary tumor location was the oral cavity in only 14.1% of the participants. Therefore, the present study findings are consistent with previous reports by Valero et al. [21] and Gürol et al. [27], who showed that high NLR was correlated with an increased risk of BSI. Additionally, the current study confirmed that elevated NLR is associated with an increased risk of BSI among elderly patients with OSCC.

Advanced tumor stage, ever betel nut chewing, and hypoalbuminemia were independent risk factors for short OS [28,29,30]. Moreover, advanced age was not associated with short OS or DFS in older patients with OSCC. Malik et al. [31] reported that age did not affect survival in patients with oral cancer. Further, advanced age may be associated with an increase in non-cancer-related mortality and comorbidities [32]. However, previous studies on OSCC have excluded patients aged > 70 years [33,34,35]; therefore, the relationship between age and survival has restricted applicability to the broader older population.

The current study observed no significant between-group differences in OS and DFS. This could be attributed to the fact that compared with the presence of sarcopenia or BSIs, advanced stage, betel nut chewing, hypoalbuminemia, and high NLR had a stronger influence on OS and DFS. According to the criteria of the European Working Group on Sarcopenia in Older People [36], it is important to examine functional measures and skeletal muscle mass in patients with sarcopenia; however, the current study defined sarcopenia only using SMI given the retrospective nature of this study and the lack of a standard criterion for defining sarcopenia in older patients with OSCC. Additionally, the current study observed no relationship of BSI with poor survival status, which could be attributed to the small sample size. The empirical antimicrobial regimen considerably influences survival in patients with BSI [37]. Moreover, only four patients had BSIs caused by multidrug-resistant strains. These factors could have contributed to the lack of an association between BSI and survival outcomes. The current study observed no differences in survival between patients who received surgery alone and adjuvant therapy. According to the NCCN guidelines [3], surgery remains the first option for elderly patients with OSCC undergoing curative-intent treatment.

When released in the tumor microenvironment, neutrophils can stimulate the migration and invasion of cancer cells; moreover, they are associated with recurrence, metastasis, and mortality in patients with OSCC [38]. A recent meta-analysis by Yang [39] showed that a high NLR was correlated with poor DFS in patients with OSCC; however, the underlying reasons were unclear. The present study assessed nutritional biomarkers, body composition, comorbidities, and inflammation biomarkers. The current study found that a high NLR was independently associated with DFS. Accordingly, it may further elucidate this issue of NLR and DFS in older patients.

This study has several limitations. First, this was a single-center retrospective study. Further large-scale cohort studies are warranted to specify the cutoff threshold for continuous biomarkers. The present study included patients with OSCC undergoing curative-intent treatment. Although the range of NLR cutoff values does not differ according to the treatment modalities for OSCC, the study findings should be interpreted with caution. Second, although the study period was > 10 years, the total number of detected BSI episodes was limited. Finally, although the study adjusted for multiple potential confounders, there might have been residual and unmeasured confounders, including lifestyle factors.

## 5. Conclusions

In conclusion, the current study found that an elevated NLR was associated with an increased risk of BSI and resistance to treatment in older patients with OSCC. Determining the NLR could allow for better assessment of the status of patients and their susceptibility to infections. Further research is warranted to determine the mechanisms underlying the relationship of neutrophil dysregulation with BSI development and resistance to treatment in older patients, which could inform future risk assessment tools and treatment decision making.

## Figures and Tables

**Table 1:** Patient characteristics.

		BSI		
Characteristics, *n* (%)	Total	Yes	No	*p*
	(*n* = 235)	(*n* = 27)	(*n* = 208)	
Age in years, median (IQR)	71.0 (67.0–75.0)	69.5 (67.7–73.0)	71.0 (67.0–76.0)	0.400
65–69	107 (45.6)	13 (48.1)	94 (45.2)	0.264
70–75	56 (23.8)	9 (33.3)	47 (22.6)	
≥76	72 (30.6)	5 (18.6)	67 (32.2)	
Sex				
Male	177 (75.3)	21 (77.8)	156 (75)	0.753
Female	58 (24.7)	6 (22.2)	52 (25)	
Alcohol				
Never	116 (49.4)	11 (40.7)	105 (50.5)	0.341
Ever	119 (50.6)	16 (59.3)	103 (49.5)	
Smoking				
Never	75 (31.9)	10 (37)	65 (31.2)	0.544
Ever	160 (68.1)	17 (63)	143 (68.8)	
Betel nut				
Never	95 (40.4)	7 (25.9)	88 (42.3)	0.103
Ever	140 (59.6)	20 (74.1)	120 (57.7)	
CCI, median (IQR)	6.0 (2.0–7.0)	6.0 (1.0–8.0)	6.0 (3.0–7.0)	0.702
<5	86 (36.6)	10 (37)	76 (36.5)	0.960
≥5	149 (63.4)	17 (63)	132 (63.5)	
Albumin				
<3.5	112 (47.7)	14 (51.9)	98 (52.9)	0.643
≥3.5	123 (52.3)	13 (48.1)	110 (47.1)	
SMI in cm^2^/m^2^, M (SD)	45.9 (8.4)	45.9 (8.4)	45.8 (8.4)	0.786
Sarcopenia				
No	70 (29.8)	6 (22.2)	64 (30.8)	0.361
Yes	165 (70.2)	21 (77.8)	144 (69.2)	
Cancer site				
Buccal mucosa	66 (28.1)	9 (33.3)	57 (27.4)	0.423
Tongue	50 (21.3)	4 (14.8)	46 (22.1)	
Lower gum	33 (14)	3 (11.1)	30 (14.4)	
Lower lip	20 (8.5)	2 (7.4)	18 (8.7)	
Retromolar trigone	15 (6.4)	1 (3.7)	14 (6.7)	
Hard palate	12 (5.1)	0 (0)	12 (5.8)	
Other sites	39 (16.6)	8 (29.7)	31 (14.9)	
Pathological stage				
I	74 (31.5)	4 (14.8)	70 (33.6)	0.212
II	59 (25.1)	7 (25.9)	52 (25)	
III	14 (6)	2 (7.4)	12 (5.8)	
IV	88 (37.4)	14 (51.9)	74 (35.6)	
Treatment type				
Surgery alone	109 (46.4)	10 (37)	99 (47.6)	0.100
Adjuvant Chemo	28 (11.9)	2 (7.4)	26 (12.5)	
Adjuvant RT	14 (6)	0 (0)	14 (6.7)	
Adjuvant CRT	84 (35.7)	15 (55.6)	69 (33.2)	
LMR, median (IQR)	4.0 (2.6–5.1)	3.6 (2.8–5.4)	4.0 (2.6–5.1)	0.292
NLR, median (IQR)	3.8 (1.7–4.8)	4.6 (2.0–5.8)	3.4 (1.7–4.5)	0.047
PLR, median (IQR)	135.6 (104.1–177.0)	143.1 (99.3–222.5)	135.5 (104.6–174.0)	0.892

BSI—bloodstream infection; CCI—Charlson comorbidity index; CRT—concurrent chemoradiotherapy; Chemo—chemotherapy; IQR—interquartile range; M—mean; RT—radiotherapy; LMR—lymphocyte-to-monocyte ratio; NLR—neutrophil-to-lymphocyte ratio; PLR—platelet-to-lymphocyte ratio; SD—standard deviation; SMI—skeletal muscle index.

**Table 2:** Distribution of pathogens isolated from blood bacterial cultures.

Variable, *n* (%)	Total	Months 1–3	Months 4–6
All pathogens	50 (100)	40 (80)	10 (20)
Gram-positive bacteria	19 (38)	15 (37.5)	4 (40)
Enterococcus faecium	1 (2)	1 (2.5)	0 (0)
VRE	1 (2)	1 (2.5)	0 (0)
Bacillus cereus	2 (4)	1 (2.5)	1 (10)
Staphylococcus aureus	6 (12)	5 (12.5)	1 (10)
ORSA	1 (2)	1 (2.5)	0 (0)
CoNS	2 (4)	1 (2.5)	1 (10)
Peptostreptococcus species	2 (4)	2 (5)	0 (0)
Viridans streptococci	4 (8)	3 (7.5)	1 (10)
Gram-negative bacteria	27 (54)	22 (55)	5 (50)
Pseudomonas aeruginosa	6 (12)	6 (15)	0 (0)
Acinetobacter baumannii	6 (12)	5 (12.5)	1 (10)
CRAB	2 (4)	2 (5)	0 (0)
Prevotella buccae	2 (4)	2 (5)	0 (0)
Enterobacter cloacae	4 (8)	1 (2.5)	3 (30)
Stenotrophomonas maltophilia	1 (2)	1 (2.5)	0 (0)
Klebsiella pneumoniae	4 (8)	3 (7.5)	1 (10)
Eikenella corrodens	1 (2)	1 (2.5)	0 (0)
Salmonella	1 (2)	1 (2.5)	0 (0)
Fungi	4 (8)	3 (7.5)	1 (10)
Candida albicans	4 (8)	3 (7.5)	1 (10)
Polymicrobial BSI	22 (44)	22 (44)	0 (0)
MDR organisms	4 (8)	4 (8)	0 (0)

CoNS—coagulase-negative staphylococcus; CRAB—carbapenem-resistant acinetobacter baumannii; ORSA—oxacillin-resistant staphylococcus aureus; VRE—vancomycin-resistant enterococci; MDR—multidrug-resistant.

**Table 3:** Logistic regression analysis for bloodstream infection.

Variable	Univariate Analysis		Multivariate Analysis	
	OR (95% CI)	*p*	OR (95% CI)	*p*
Age ≥ 65	0.97 (0.48–1.28)	0.346		
Alcohol				
Never	1 [reference]			
Ever	1.48 (0.65–3.34)	0.343		
Smoking				
Never	1 [reference]			
Ever	0.77 (0.33–1.78)	0.545		
Betel nut				
Never	1 [reference]			
Ever	2.09 (0.84–5.17)	0.109		
CCI				
<5	1 [reference]			
≥5	0.97 (0.42–2.24)	0.960		
Albumin				
≥3.5	1 [reference]			
<3.5	2.02 (0.83–4.91)	0.118		
Sarcopenia				
No	1 [reference]			
Yes	1.55 (0.59–4.03)	0.364		
Pathological stage				
I-II	1 [reference]			
III- IV	2.06 (0.91–4.66)	0.082		
Treatment type				
Surgery alone	1 [reference]		1 [reference]	
Adjuvant Chemo	0.52 (0.12–2.79)	0.499	0.49 (0.09–2.57)	0.404
Adjuvant CRT	2.43 (1.02–5.76)	0.043	2.00 (0.80–5.03)	0.137
LMR				
<4.0	1 [reference]			
≥4.0	1.40 (0.58–3.35)	0.448		
NLR				
<5	1 [reference]		1 [reference]	
≥5	9.17 (2.37–9.86)	<0.001	9.78 (4.14–9.99)	<0.001
PLR				
<135.6	1 [reference]			
≥135.6	1.31 (0.56–3.06)	0.522		

CCI—Charlson comorbidity index; CRT—concurrent chemoradiotherapy; Chemo—chemotherapy; CI—confidence interval; LMR—lymphocyte-to-monocyte ratio; NLR—neutrophil-to-lymphocyte ratio; OR—odds ratio; PLR—platelet-to-lymphocyte ratio.

**Table 4:** Univariate analysis of prognostic factors of overall survival and disease-free survival using Cox regression model.

Variable	Overall Survival		Disease-Free Survival	
	HR (95% CI)	*p*	HR (95% CI)	*p*
Age ≥ 65	1.03 (0.98–1.08)	0.237	1.01 (0.97–1.04)	0.455
Alcohol				
Never	1 [reference]		1 [reference]	
Ever	1.31 (0.78–2.19)	0.294	1.17 (0.82–1.67)	0.359
Smoking				
Never	1 [reference]		1 [reference]	
Ever	1.02 (0.59–1.76)	0.928	1.05 (0.71–1.54)	0.798
Betel nut				
Never	1 [reference]		1 [reference]	
Ever	2.03 (1.14–3.59)	0.015	1.46 (1.01–2.12)	0.044
CCI				
<5	1 [reference]		1 [reference]	
≥5	1.64 (0.85–3.18)	0.136	0.89 (0.60–1.31)	0.566
Albumin				
≥3.5	1 [reference]		1 [reference]	
<3.5	2.66 (1.31–5.40)	0.006	1.56 (0.92–2.64)	0.096
Sarcopenia				
No	1 [reference]		1 [reference]	
Yes	1.51 (0.91–2.51)	0.107	1.33 (0.93–1.90)	0.109
Pathological stage				
I-II	1 [reference]		1 [reference]	
III-IV	2.18 (1.31–3.62)	0.003	1.45 (1.02–2.07)	0.038
Treatment type				
Surgery alone	1 [reference]		1 [reference]	
Adjuvant Chemo	1.68 (0.82–3.45)	0.151	1.53 (0.90–2.60)	0.108
Adjuvant RT	1.32 (0.39–4.42)	0.646	1.49 (0.67–3.30)	0.324
Adjuvant CRT	1.68 (0.94–2.99)	0.077	1.41 (0.94–2.10)	0.090
BSI				
No	1 [reference]		1 [reference]	
Yes	2.03 (1.05–3.92)	0.033	1.32 (0.78–2.20)	0.290
LMR				
<4.0	1 [reference]		1 [reference]	
≥4.0	1.22 (0.73–2.05)	0.433	1.18 (0.82–1.71)	0.357
NLR				
<2.9	1 [reference]		1 [reference]	
≥2.9	1.14 (1.04–1.24)	0.004	1.63 (1.11–2.39)	0.012
PLR				
<135.6	1 [reference]		1 [reference]	
≥135.6	1.31 (0.76–2.23)	0.323	1.00 (0.68–1.45)	0.997

BSI—bloodstream infection; CCI—Charlson comorbidity index; CRT—concurrent chemoradiotherapy; Chemo—chemotherapy; CI—confidence interval; HR—hazard ratio; LMR—lymphocyte-to-monocyte ratio; NLR—neutrophil-to-lymphocyte ratio; PLR—platelet-to-lymphocyte ratio; RT—radiotherapy.

**Table 5:** Multivariate analysis of prognostic factors of overall survival and disease-free survival using Cox regression model.

Variable	Overall Survival		Disease-Free Survival	
	HR (95% CI)	*p*	HR (95% CI)	*p*
Betel nut				
Never	1 [reference]		1 [reference]	
Ever	2.99 (1.11–8.05)	0.016	0.69 (0.47–1.03)	0.071
Albumin				
≥3.5	1 [reference]			
<3.5	2.32 (1.11–4.83)	0.024		
Pathological stage				
I-II	1 [reference]		1 [reference]	
III-IV	2.32 (1.08–4.99)	0.031	1.25 (0.84–1.84)	0.262
BSI				
No	1 [reference]			
Yes	1.12 (0.41–3.06)	0.818		
NLR				
<2.9	1 [reference]		1 [reference]	
≥2.9	1.39 (0.64–3.01)	0.404	1.55 (1.060–2.29)	0.024

BSI—bloodstream infection; CCI—Charlson comorbidity index; CRT—concurrent chemoradiotherapy; Chemo—chemotherapy; CI—confidence interval; HR—hazard ratio; LMR—lymphocyte-to-monocyte ratio; NLR—neutrophil-to-lymphocyte ratio; PLR—platelet-to-lymphocyte ratio; RT—radiotherapy.

## Data Availability

The data presented in this study are available on request from the corresponding author. The data are not publicly available due to privacy and ethical restrictions.

## Supplementary Materials

The following are available online at https://www.mdpi.com/article/10.3390/diagnostics13030493/s1, Table S1: Details regarding chemotherapy regimen; Table S2: Receiver operating characteristic curve analysis for bloodstream infection; Table S3: Time-dependent receiver operating characteristic curve analysis for overall and disease-free survival; Figure S1: Study flow chart.
