# Supplementary material for: Association of Neutrophil-to-Lymphocyte Ratio and Bloodstream Infections with Survival after Curative-Intent Treatment in Elderly Patients with Oral Cavity Squamous Cell Carcinoma
Source: Diagnostics (Basel). 2023 Jan 29;13(3):493. doi: 10.3390/diagnostics13030493 (PMC9914317; doi:10.3390/diagnostics13030493)
Supplement: Supplementary file 1 [file diagnostics-13-00493-s001.zip › Diagnostics_OSCC_Supplementary Online Content 0128-2320.pdf]

Supplementary Online Content

Chun-Hou Huang, Yu-Fu Chou, Tsung-Cheng Hsieh and Peir-Rong Chen. Association of Neutrophil-to-Lymphocyte Ratio and Blood-stream Infections with Survival after Curative-Intent Treatment in Elderly Patients with Oral Cavity Squamous Cell Carcinoma. *Diagnostics*

Table S1: Details regarding chemotherapy regimen

Table S2: Receiver operating characteristic curve analysis for bloodstream infection

Table S3: Time-dependent receiver operating characteristic curve analysis for overall and disease-free survival

Figure S1: Study flow chart

This supplementary material has been provided by the authors to give readers additional information about their work.

Table S1: Details regarding chemotherapy regimen

Variables	Total	BSI	
		Yes	No
**Adjuvant chemotherapy, n (%)**	28 (100)	2 (7.1)	26 (92.9)
Tegafur-uracil	24 (85.7)	0 (0)	24 (92.3)
Cisplatin	4 (14.3)	2 (100)	2 (7.7)
**Adjuvant CRT, n (%)**	84 (100)	15 (17.9)	69 (82.1)
Tegafur-uracil	56 (66.7)	10 (66.7)	46 (66.7)
Cisplatin	23 (27.4)	4 (26.7)	19 (27.5)
PF	5 (5.9)	1 (6.6)	4 (5.8)

BSI, bloodstream infection; CRT, concurrent chemoradiotherapy; PF, cisplatin plus 5-FU.

Table S2: Receiver operating characteristic curve analysis of factors related to bloodstream infection

	Bloodstream infection		
Parameter	LMR	NLR	PLR
AUC	0.542	0.691	0.538
Optimal sensitivity (%)	0.521	0.733	0.520
Optimal specificity (%)	0.594	0.604	0.642
Maximizes the sum of sensitivity and specificity	3.65	5	149.7

AUC: area under curve; LMR: lymphocyte-to-monocyte ratio; NLR: neutrophil-to-lymphocyte ratio; PLR: platelet-to-lymphocyte ratio.

Table S3: Time-dependent receiver operating characteristic curve analysis of factors related to overall and disease-free survival

Parameter	Overall survival			Disease-free survival		
	LMR	NLR	PLR	LMR	NLR	PLR
AUC	0.461	0.651	0.55	0.386	0.634	0.523
Optimal sensitivity (%)	0.141	0.614	0.806	0.125	0.69	0.748
Optimal specificity (%)	0.945	0.68	0.355	1	0.664	0.39
Maximizes the sum of sensitivity and specificity	6.4	2.9	108.56	6.4	2.9	139.3

AUC: area under curve; LMR: lymphocyte-to-monocyte ratio; NLR: neutrophil-to-lymphocyte ratio; PLR: platelet-to-lymphocyte ratio.

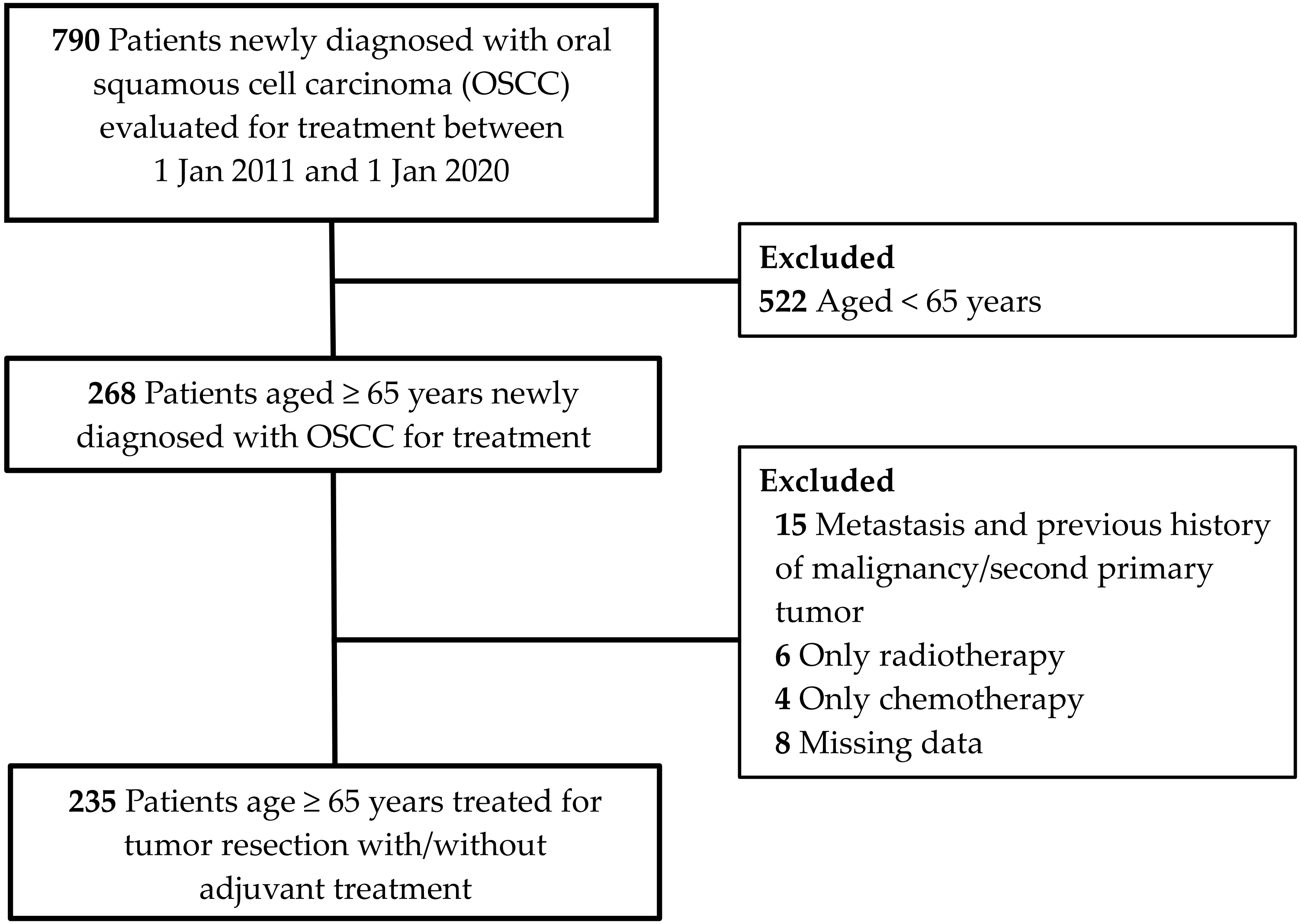

Figure S1: Study flow chart
